# Orthostatic intolerance syndromes after hematopoietic cell transplantation: clinical characteristics and therapeutic interventions in a single-center experience

**DOI:** 10.1186/s40959-021-00126-7

**Published:** 2021-11-30

**Authors:** Alessandra Vecchié, Georgia Thomas, Edoardo Bressi, Aldo Bonaventura, Justin M. Canada, David Chuquin, Dinesh Kadariya, Usman Piracha, Delia Endicott, Roshanak Markley, Amir Toor, Michael Hess, Antonio Abbate

**Affiliations:** 1 VCU Pauley Heart Center, Virginia Commonwealth University, 1200 E. Broad Street, Box 980204, Richmond, VA 23298 USA; 2 Department of Internal Medicine, Ospedale di Circolo e Fondazione Macchi, ASST Sette Laghi, Varese, Italy; 3 First Clinic of Internal Medicine, Department of Internal Medicine, University of Genoa, 6 viale Benedetto XV, 16132 Genoa, Italy; 4 Massey Cancer Center, Virginia Commonwealth University, Richmond, USA

**Keywords:** Dysautonomia, Orthostatic hypotension, Postural tachycardia syndrome, Bone marrow transplant, Chemotherapy

## Abstract

**Background:**

Hematopoietic cell transplantation (HCT) is an established and potentially curative therapeutic option for hematologic cancers. HCT survivors are at risk of developing long-term complications impacting on morbidity and mortality. Orthostatic hypotension (OH) and postural tachycardia syndrome (POTS) have been anecdotally described after HCT. However, the incidence and clinical characteristics of patients with OH and POTS after HCT has not been well defined.

**Methods:**

This retrospective study included 132 patients who had HCT between March 2011 and July 2018 and were referred to Cardio-oncology clinic. Patients were screened for OH and POTS. Using logistic regression analysis we evaluated the association between clinical factors and the incidence of OH and POTS.

**Results:**

Median age was 58 (47–63) years, 87 (66%) patients were male, 95 (72%) were Caucasian. OH was diagnosed in 30 (23%) subjects and POTS in 12 (9%) after the HCT. No significant differences in demographic characteristics were found when comparing patients with and without OH or POTS. The two groups did not differ for cardiovascular diseases prevalence nor for the prior use of antihypertensive drugs. Previous radiotherapy and treatment with specific chemotherapy drugs were found to be associated with the incidence of OH or POTS, but none of the factors maintained the significance in the multivariate model. Pharmacological therapy was required in 38 (91%) cases, including a b-adrenergic blocker (*n* = 24, 57%), midodrine (n = 24, 57%) and fludrocortisone (*n* = 7, 18%).

**Conclusion:**

Orthostatic intolerance syndromes are commonly diagnosed in patients referred to the cardiologist after HCT, involving approximately 1/3 of patients and requiring pharmacological therapy to cope with symptoms in the majority of cases. Risk factors specific to this population are identified but cannot fully explain the incidence of POTS and OH after HCT.

**Supplementary Information:**

The online version contains supplementary material available at 10.1186/s40959-021-00126-7.

## Background

Hematopoietic cell transplantation (HCT) is the treatment of choice for several hematologic malignancies. Advances in HCT techniques and post-transplant treatments have improved the survival of these patients considerably [1]. Long-term survivors, however, are at risk for developing treatment-related complications. HCT survivors have a 0.6 to 5.6-fold increased risk of cardiovascular (CV) diseases (CVD) including coronary artery disease, cerebrovascular disease, and heart failure [2]. In patients with Hodgkin’s disease that had received mediastinal irradiation, persistent tachycardia and a blunted hemodynamic response to exercise were observed in nearly 1/3 of patients [3]. General autonomic dysfunction was found in 50–63% of patients with advanced cancer and may be predictive of reduced survival [4].

Orthostatic intolerance syndromes, which include orthostatic hypotension (OH) and postural tachycardia syndrome (POTS), are a constellation of conditions characterized by the inability to tolerate the upright position as a consequence of structural or functional autonomic nervous system failure, which impairs the CV compensatory mechanisms usually activated after standing [5]. They are often under recognized or misdiagnosed, and their prevalence is higher than usually thought, both in the general population and in specific diseases. An increased rate of CV events and subclinical CVD have been recently seen in patients with OH compared to general population [6]. OH has been reported in a considerable number of patients after lung and kidney-pancreas transplants [7, 8], although the causes remain unclear. The incidence of OH and POTS after HCT, instead, has not yet been described.

In the current study, we aimed to evaluate the occurrence of OH and POTS in a cohort of patients who were referred to the cardio-oncology clinic for evaluation after HCT for hematologic malignancies to gain insight into clinical features that may be associated with the development of these conditions.

## Methods

### Study design and definitions

In this retrospective study we included 132 consecutive patients aged 18 years or older who had HCT for a hematologic malignancy at Virginia Commonwealth University Medical Center between March 2011 and July 2018 and were referred to Cardio-oncology clinic for the evaluation before the procedure. The de-identified medical records were reviewed to obtain demographic data and past medical history including underlying hematologic malignancy, date and type of HCT, pre-transplant CV risk factors and CVD. After HCT, the patients were followed by a Cardiologist. Supine, sitting and standing blood pressure (BP) and heart rate (HR) were measured during the visits. BP and HR were measured according to the American Heart Association recommendations [9]. All subjects were allowed to rest for 10 min, were pain free and allowed to urinate if needed. BP and HR were measured supine first, then 1 min after changing to the sitting position, then 1 min after changing to the standing position, and when possible the subject was maintained standing for up to 10 min with BP and HR measured at 3, 5, 7 and 10 min and the last values measured were used.

OH was defined as a drop in systolic BP ≥20 mmHg and or in diastolic BP ≥10 mmHg within 3 min of active standing [10, 11]. POTS was identified by an increase in HR of more than 30 beats per minute within 10 min of active standing, without a drop in BP values [10, 11]. All the patients diagnosed with OH or POTS had symptoms of orthostatic intolerance, such as light-headedness, weakness, blurred vision, etc. At least two physicians reviewed patient’s charts to identify those who had developed OH or POTS after HCT. Disagreements on findings were resolved by discussion.

The study was conducted in accordance with the Declaration of Helsinki and approved by the local Institutional Review Board.

### Study endpoints

The primary endpoint of this study was to determine the incidence of OH and POTS after HCT. Secondary aims include the identification of clinical factors associated with the development of OH and POTS and the analysis of the treatments started for these conditions.

### Statistical analysis

Continuous variables were presented as median and interquartile range, and data were compared using Mann Whitney *U* test. Categorical values were expressed as absolute and relative frequencies, and data were compared with χ^2^ test or Fisher’s test, as appropriate. For the logistic regression analysis, the measure of uncertainty was expressed as an odds ratio (OR) and 95% confidence interval (CI). Treatments received by patients before the transplant were selected as the variables for the logistic regression analysis.

For all statistical analyses, a two-sided *p* < 0.05 value was considered statistically significant. Statistical analyses were performed with IBM SPSS Statistics for Windows, Version 25.0 (IBM CO., Armonk, NY) and GraphPad Prism, version 8.2 for Windows (GraphPad Software, La Jolla, CA, USA, www.graphpad.com).

## Results

### Clinical characteristics of the cohort

Demographic and clinical characteristics of the cohort are summarized in Table 1. Patients’ median age was 58 (47–63) years, with 66% (*n* = 87) of male and 72% (*n* = 90) of self-defined Caucasians. Hypertension was the most common CVD before the transplant (71 patients, 54%). Atrial fibrillation and heart failure were observed in 33 (25%) and 28 (21%) subjects, respectively. A significant number of patients were receiving treatment with anti-hypertensive drugs at the time of follow-up with the cardiologist (Table 1).

**Table 1:** Characteristics of the overall cohort

	Patients (***n*** = 132)
Age, years	58 [47–63]
Male sex	87 (65.9)
Race	
Black	33 (25.0)
White	95 (72.0)
Other	4 (3.0)
Hematologic diagnosis	
Multiple myeloma	32 (24.2)
Myelodysplastic syndrome	9 (6.8)
Myelofibrosis	9 (6.8)
Acute myeloid leukemia	27 (20.5)
Acute lymphatic leukemia	11 (8.3)
Chronic myeloid leukemia	3 (2.3)
Chronic lymphatic leukemia	2 (1.5)
Hodgkin’s lymphoma	8 (6.1)
Non-Hodgkin’s lymphoma	20 (15.2)
Aplastic anemia	4 (3.0)
Other	7 (5.4)
Donor status	
Related	28 (21.2)
Unrelated	55 (41.7)
Not applicable	49 (37.1)
HCT type	
Autologous	49 (37.1)
Allogenic	83 (62.9)
Cardiovascular diseases and risk factors	
Hypertension	71 (53.8)
Smoking	3 (2.3)
Diabetes mellitus	20 (15.2)
Hypercholesterolemia	35 (26.5)
Chronic kidney disease	4 (3.0)
CAD	14 (10.3)
Heart failure	28 (21.2)
Atrial fibrillation	33 (25.0)
Pericarditis	6 (4.5)
None	25 (18.9)
Anti-hypertensive drugs prior to HCT	
b-blockers	68 (51.5)
Calcium-channel blockers	29 (22.0)
ACE inhibitors	16 (12.1)
ARBs	6 (4.5)
Furosemide/Torsemide	21 (15.9)
Spironolactone	8 (6.1)
None	92 (69.7)
Orthostatic Intolerance Syndromes	47 (34.6)
Orthostatic hypotension pre-HCT	3 (2.3)
Orthostatic hypotension post-HCT	30 (22.7)
Tachycardia syndrome	12 (9.1)

The data are presented as a number and (%) of all cases or as median and [interquartile range]

*Abbreviations*: *ACE* Angiotensin-converting enzyme, *ARBs* Angiotensin II receptor blockers, *CAD* Coronary artery disease, *HCT* Hematopoietic cell transplantation

Allogenic transplants constituted 63% (*n* = 83) of HCT (Table 1). HCT was performed in 34 patients (24%) to treat multiple myeloma, in 27 (21%) for acute myeloid leukemia and in 20 (15%) for non-Hodgkin’s lymphoma. Other less frequent hematologic diagnoses are listed in Table 1. Before the transplant, 93% of patients (*n* = 123) were treated with alkylating agents. Antimetabolites drugs were used in 67% of subjects (*n* = 89) and anthracyclines in 43% of cases (*n* = 57). Radiotherapy was required in 35 patients (27%). The other class of chemotherapy/immunotherapy drugs used in our cohort are illustrated in Supplemental Fig. 1 and a complete list of agents is provided in the Supplementary Table 1.

Among the patients who underwent allogenic transplants, 30 (37%) presented graft-versus-host disease (GVHD) at the time of the cardiologic evaluation post-HCT. The GVHD was acute in 24 (80%) cases and chronic in 6 (20%).

### Orthostatic intolerance syndromes

The presence of an orthostatic intolerance syndrome was recorded in 45 patients (34%): 12 (9%) had POTS and 33 (25%) had OH. Among them, three subjects had OH before transplant. The median time from HCT to the diagnosis of new onset of OH or POTS was 82 (24–248) days. In the group of patients who developed OH after the HCT, nine had supine hypertension. No significant differences in baseline demographic characteristics were found when comparing patients with and without OH or POTS (Table 2), and neither the hematologic diagnosis nor transplant type differed between two groups. The prevalence of CV diseases and risk factors was similar in patients who developed OH or POTS compared to those without dysautonomia, and the percentage of subjects treated with antihypertensive drugs did not differ in the two groups.

**Table 2:** Characteristics of the overall cohort based on the occurrence of orthostatic hypotension or tachycardia syndrome after bone marrow transplant

	Patients without OH/POTS (***n*** = 89)	Patients with OH/POTS (***n*** = 47)	***p***
Age, years	58 [49–63]	55 [44–63]	0.26
Male sex	57 (63.3)	30 (71.4)	0.43
Race			
Black	24 (26.7)	9 (21.4)	0.15
White	64 (72.2)	30 (71.4)	
Other	1 (1.1)	3 (7.1)	
Hematologic diagnosis			
Multiple myeloma	22 (24.4)	10 (23.8)	0.40
Myelodysplastic syndrome	7 (7.8)	2 (4.8)	
Myelofibrosis	7 (7.8)	2 (4.8)	
Acute myeloid leukemia	19 (21.1)	8 (19.0)	
Acute lymphatic leukemia	6 (6.7)	5 (11.9)	
Chronic myeloid leukemia	2 (2.2)	1 (2.4)	
Chronic lymphatic leukemia	2 (2.2)	0	
Hodgkin’s lymphoma	5 (5.6)	3 (7.1)	
Non-Hodgkin’s lymphoma	12 (13.3)	8 (19.0)	
Aplastic anemia	4 (4.4)	0	
Other	4 (4.4)	3 (7.2)	
Donor status			
Related	19 (21.1)	9 (21.4)	0.83
Unrelated	39 (43.3)	16 (38.1)	
BMT type			
Autologous	32 (35.6)	17 (40.5)	0.7
Allogenic	58 (64.4)	25 (59.5)	
CV diseases and risk factors			
Hypertension	53 (58.9)	18 (42.9)	0.1
CAD	11 (12.2)	3 (7.1)	0.55
Heart failure	22 (24.4)	6 (14.3)	0.25
Diabetes mellitus	16 (17.8)	4 (9.5)	0.30
Hypercholesterolemia	25 (27.8)	10 (23.8)	0.68
Atrial fibrillation	25 (27.8)	8 (19.0)	0.39
Chronic kidney disease	2 (2.2)	2 (4.8)	0.59
Smoking	2 (2.2)	1 (2.4)	1.00
Pericarditis	4 (4.4)	2 (4.8)	1.00
Treatment with anti-hypertensive drugs prior to BMT			
b-adrenergic blockers	46 (51.1)	22 (52.4)	1.00
Calcium-channel blockers	21 (23.3)	8 (19.0)	0.66
ACE inhibitors	14 (15.6)	2 (4.8)	0.09
ARBs	4 (4.4)	2 (4.8)	1.00
Furosemide/Torsemide	18 (20.0)	3 (7.1)	0.08
Spironolactone	5 (5.6)	3 (7.1)	0.71
None	24 (27%)	16 (38)	0.22
Number of anti-hypertensive drugs prior to BMT			
1	37 (41.1)	14 (33.3)	0.39
2	17 (18.9)	10 (23.8)	
3	11 (12.2)	2 (4.8)	
4	1 (1.1)	1 (1.1)	

The data are presented as a number and (%) of all cases or as median and [interquartile range]. *P*-values were calculated by Kruskall Wallis or Fisher’s exact test or χ^2^ test, as appropriate. Statistical significant values are presented in bold character

*Abbreviations*: *ACE* Angiotensin-converting enzyme, *ARBs* Angiotensin II receptors blockers, *CAD* Coronary artery disease, *CV* Cardiovascular, *HCT* Hematopoietic cell transplantation, *OH* Orthostatic hypotension, *POTS* Postural orthostatic tachycardia syndrome

**Fig. 1:**
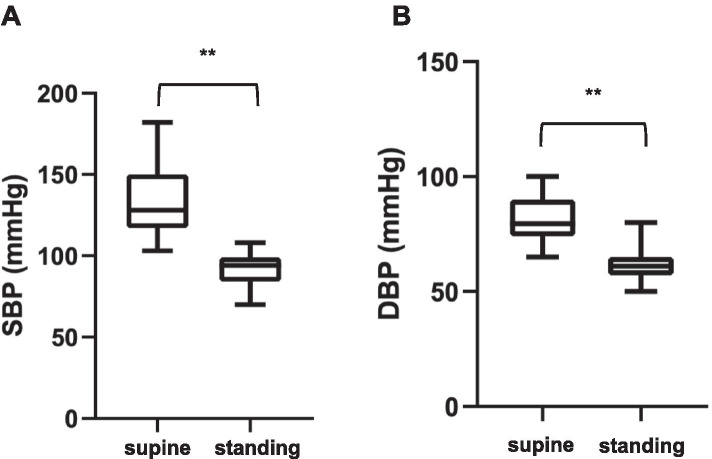
Differences of SBP and DBP from supine to standing position in patients with orthostatic hypotension at the time of the cardiologic visit. Boxplot showing the SBP and DBP values in the supine and in the standing position. Data are showed as minimum to maximum with the midline representing the median. ** *p* < 0.001

**Fig. 2:**
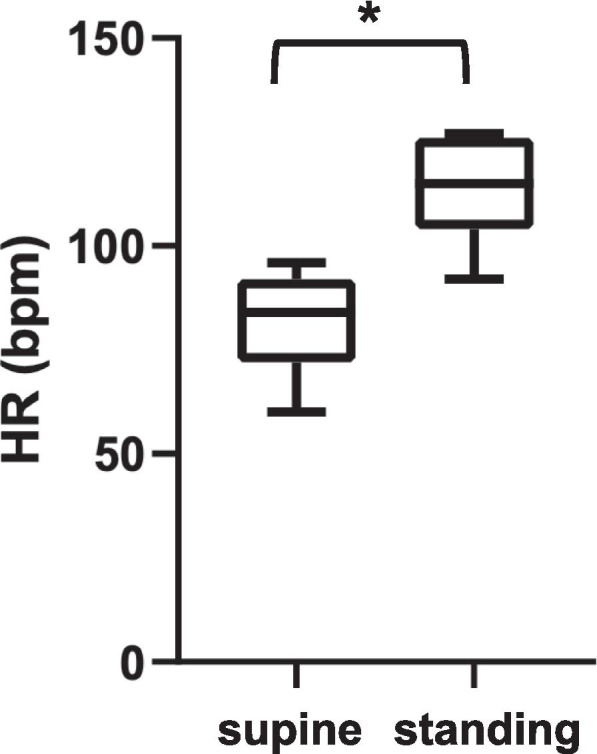
Differences of HR from supine to standing position in patients with postural orthostatic tachycardia syndrome at the time of the cardiologic visit. Boxplot showing the HR values in the supine and in the standing position. Data are showed as minimum to maximum with the midline representing the median. ** p < 0.001

The characteristic of patients who developed OH or POTS are summarized in Table 3. The median age was 57 (47–65) years in patients with OH and 50 (39–58) in those with POTS. Male sex and white race were prevalent in both groups. In a subgroup of patients (*n* = 16) with OH/POTS only semi-quantitative descriptions were available >20 mmHg drop with standing” or “>30 beats per minute increase with standing”.) The BP values in clinostatism were available for 19 patients in the group diagnosed with OH. The systolic BP dropped from supine to standing position (128 [117–150] vs 92 [83–99], *P* < 0.001) as well as the diastolic BP (80 [74–90] vs 61 [57–64], P < 0.001) (Fig. 1). HR values in clinostatism were available for 7 patients in the subgroup diagnosed with POTS and a significant increased from supine to standing position was observed (84 [72–92] vs 115 [104–126], *P* = 0.01) (Fig. 2). Factors that would predict OH rather than POTS could not be identified (Table 3).

**Table 3:** Characteristics of patients who developed orthostatic hypotension or postural tachycardia syndrome after HCT

	Patients with OH (***n*** = 30)	Patients with POTS (***n*** = 12)	***P***-value
Age, years	57 [47–65]	50 [39–58]	0.1
Male sex	20 (66.7)	10 (83.3)	0.45
Race			
African-American	8 (26.7)	1 (8.3)	0.43
White	20 (66.7)	10 (83.3)	
Other	2 (6.7)	1 (8.3)	
Hematologic diagnosis			
Multiple myeloma	6 (20.0)	4 (33.3)	0.67
Myelodysplastic syndrome	1 (3.3)	1 (8.3)	
Myelofibrosis	1 (3.3)	1 (8.3)	
Acute myeloid leukemia	5 (16.7)	3 (25.0)	
Acute lymphatic leukemia	4 (13.3)	1 (8.3)	
Chronic myeloid leukemia	1 (3.3)	0	
Chronic lymphatic leukemia	0	0	
Hodgkin’s lymphoma	3 (10.0)	0	
Non-Hodgkin’s lymphoma	6 (20.0)	2 (16.7)	
Aplastic anemia	0	0	
Other	3 (9.9)	0	
Donor status			
Related	7 (23.3)	2 (16.7)	0.61
Unrelated	10 (33.3)	6 (50.0)	
BMT type			
Autologous	13 (43.3)	4 (33.3)	0.73
Allogenic	17 (56.7)	8 (66.7)	
CV diseases and risk factors			
Hypertension	12 (40.0)	6 (50.0)	0.73
CAD	1 (3.3)	2 (16.7)	0.19
Heart failure	5 (16.7)	1 (8.3)	0.66
Diabetes mellitus	1 (3.3)	3 (25.0)	0.06
Hypercholesterolemia	5 (16.7)	5 (41.7)	0.12
Atrial fibrillation	5 (16.7)	3 (25.0)	0.69
Chronic kidney disease	1 (3.3)	1 (8.3)	0.5
Smoking	0	1 (8.3)	0.27
Pericarditis	1 (3.3)	1 (8.3)	0.5
Previous treatment with anti-hypertensive drugs			
b-blockers	14 (46.7)	8 (66.7)	0.32
Calcium-channel blockers	5 (16.7)	3 (25.0)	0.67
ACE inhibitors	1 (3.3)	1 (8.3)	0.5
ARBs	1 (3.3)	1 (8.3)	0.5
Furosemide/Torsemide	2 (6.7)	1 (8.3)	1.00
Spironolactone	1 (3.3)	2 (16.7)	0.19
None	14 (46.7)	2 (16.7)	0.09
Number of anti-hypertensive drugs			
1	9 (30.0)	5 (41.7)	0.1
2	6 (20.0)	4 (33.3)	
3	1 (3.3)	1 (8.3)	
4	1 (3.3)	0	

The data are presented as a number and (%) of all cases or as median and [interquartile range]

*Abbreviations*: *ACE* Angiotensin-converting enzyme, *ARBs* Angiotensin II receptors blockers, *CAD* Coronary artery disease, *CV* Cardiovascular, *HCT* Hematopoietic cell transplantation, *OH* Orthostatic hypotension, *POTS* Postural orthostatic tachycardia syndrome

In a logistic regression model, radiotherapy was associated with a significant risk of OH or POTS development after HCT (OR 2.682, CI 95% 1.200–5.996, *p*-value 0.02) (Table 4). Treatment with specific antineoplastic medications was also found to be associated with OH or POTS occurrence: anthracyclines (OR 2.303, CI 95% 1.092–4.859, p-value 0.03), corticosteroids (OR 2.364, CI 95% 1.104–5.060, p-value 0.03), anti-microtubules drugs (OR 2.891, CI 95% 1.319–6.339, p-value 0.008), and epipodophyllotoxin (OR 2.850, CI 95% 1.299–6.251, p-value < 0.01) (Table 4). When these treatments were included in a multivariate logistic regression model, however, none of them maintained statistical significance.

**Table 4:** Logistic regression analysis showing the association between therapy and OH or POTS at univariate and multivariate analysis

	Univariate model			Multivariate model		
	OR	95% CI	*P*-value	OR	95% CI	*P*-value
Radiotherapy	**2.68**	**1.20–6.0**	**0.02**	1.92	0.8–4.61	0.15
Anthracyclines	**2.30**	**1.09–4.86**	**0.03**	1.26	0.45–3.52	0.66
Corticosteroids	**2.36**	**1.10–5.06**	**0.03**	1.5	0.62–3.6	0.37
Anti-microtubules	**2.89**	**1.32–6.34**	**0.01**	1.15	0.32–4.12	0.83
Epipodophyllotoxin	**2.85**	**1.3–6.25**	**0.01**	1.90	0.71–5.1	0.20
Angiogenesis inhibitor	0.9	0.37–2.17	0.81			
Histone-deacetylase inhibitors	0.42	0.05–3.67	0.43			
Kinase inhibitors	1.09	0.40–2.93	0.87			
Antimetabolites	0.95	0.44–2.07	0.9			
Antitumor antibiotics	1.89	0.54–6.59	0.32			
Proteasome inhibitors	0.87	0.38–1.99	0.75			
Anti-thymocyte globulin	0.72	0.34–1.52	0.39			
Alkylating agents	0.93	0.22–3.91	0.92			

Statistically significant *p* values are displayed in bold characters

*CI* Confidence interval, *OH* Orthostatic hypotension, *POTS* Postural orthostatic tachycardia syndrome

### Treatment for orthostatic hypotension and postural orthostatic tachycardia syndrome

**Fig. 3:**
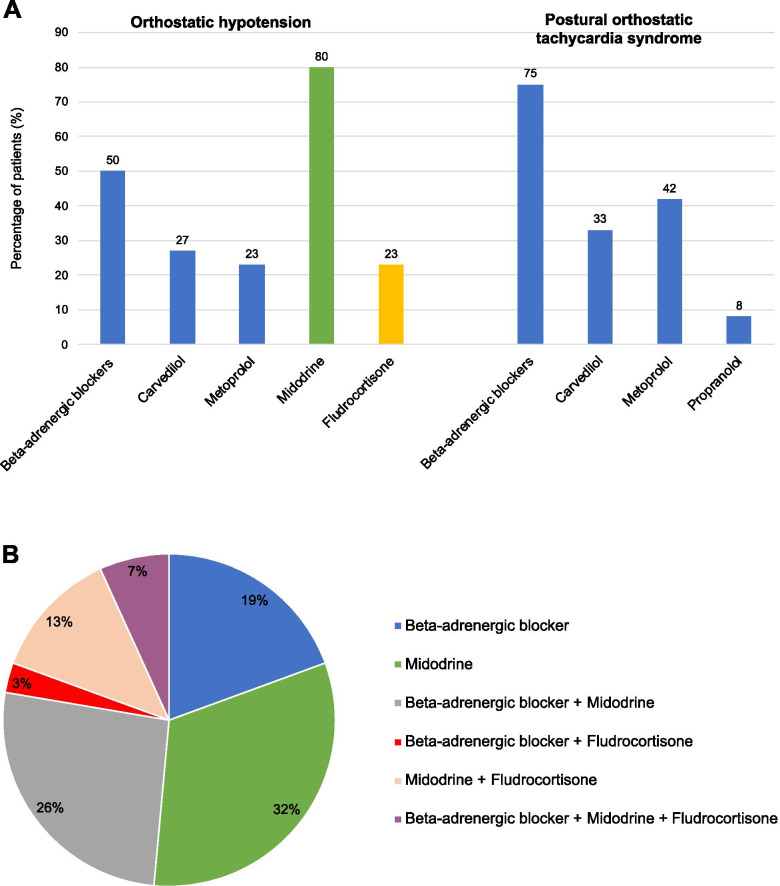
**A** Pharmacological therapies in patients who developed OH and POTS after HTC. The column graphic represents the percentage of patients treated with different classes of drugs. **B** Combination of pharmacological treatments in patients who developed OH after HCT. The pie chart represents the percentage of patients treated with different combinations of drugs HTC: hematopoietic cell transplantation; OH: orthostatic hypotension; POTS: postural orthostatic tachycardia syndrome.

All patients diagnosed with OH or POTS received education on non-pharmacological treatments which include: 1) avoidance of triggering situations (such as prolonged standing motionless and recumbency, sudden changes in posture, consumptions of alcohol and large meals); 2) elastic stockings; 3) maintenance of adequate hydration and salt intake; 4) exercise. Pharmacological therapy was prescribed in patient with persistent symptoms despite the adoption of these behavioral modifications (*n* = 38, 91%)(Fig. 3A). In the group of 12 patients with POTS, a b-adrenergic blocker was prescribed to 9 (75%) patients, metoprolol in 5 (42%) cases, carvedilol in 4 (33%) and propranolol in 1 (8%). In the group of 30 patients with OH, 24 (80%) subjects received midodrine, 15 (50%) a b-blocker and 7 (23%) fludrocortisone (Fig. 3A). Of these, 16 (51%) patients were treated with only 1 drug, b-adrenergic blocker in 6 (20%) and midodrine in 10 (33%). A combination of a b-adrenergic blocker and midodrine was used in 8 (27%) patients, and 4 (13%) patients were treated with midodrine and fludrocortisone, 1 (3%) patient received a b-adrenergic blocker in association with fludrocortisone, and 2 (7%) required a combination of a b-adrenergic blocker, midodrine and fludrocortisone to treat the symptoms of OH (Fig. 3B).

All patients described a favorable response to treatment: 17 (45%) patients had resolution of symptoms on monotherapy, the remaining 18 (55%) patients required 2 or more therapies. Of those with OH, midodrine was used as first-line therapy in 26 (87%) patients, and fludrocortisone was added in 8 (27%) cases, hydrocortisone in 1 (3%), and β-adrenergic blockers in 5 (17%) cases. Treatment was interrupted after a median duration of 120 (33–333) days in 21 (60%) of cases.

## Discussion

Orthostatic intolerance syndromes, such as OH and POTS, affect both the general population and patients with specific diseases, including those who have received organ transplants [7, 8, 12]. Orthostatic intolerance syndromes are associated with an increased risk of CVD and higher mortality [6, 12] and with a diminished quality of life, emphasizing the need for better recognition. In the recent years, autonomic dysfunction has become a recognized CV disorder affecting patients with cancer and cancer survivors more than healthy controls [13].

In the current study, we described for the first time the prevalence of OH and POTS in patients referred to the cardio-oncology clinic following HCT for several hematologic disorders. OH occurred in 23% of patients and POTS in 9% after HCT. In this cohort of patients, the development of OH or POTS was not associated with the risk factors previously described in other populations, such as age, sex, and the use of anti-hypertensive drugs. While radiotherapy and the use of specific antineoplastic regimens may be risk factors for the development of OH and POTS, the occurrence of these syndromes is more likely associated with the transplant procedure by itself, considering that patients had not been diagnosed with these prior to HCT and none of these risk factors were significant in the multivariate analysis.

Prevalence of OH similar to that found in our population has been previously described by Schuurmans et al. in a small group of lung transplant recipients [8]. In another recent study, OH occurred in 28% of kidney-pancreas transplant patients [7]. History of peripheral neuropathy related to long-standing diabetes, was not correlated with the development of OH in this group of patients [7]. Hyperinsulinemia after transplant and vasodilator neuropeptides released from the pancreas were described as possible responsible agents for OH development, but the biological cause remains unclear.

Berger et al. reported a higher presence of dysautonomia in a group of childhood cancer survivors compared with healthy matched controls [14]. No differences in factors possibly causing dysautonomia were found between the two groups, but authors were not able to examine a possible association with cancer treatment due to the small sample size [14]. Recently, in a study by Deuring et al., cardiac autonomic function was measured in a group of HCT survivors of and in healthy matched controls, by the assessment of HR and respiratory sinus arrhythmia (RSA) [15]. Transplant recipients showed a higher HR and lower RSA compared to control group, indicating the presence of cardiac autonomic dysfunction [15].

In the general population the incidence of OH exponentially increase with the age, not unexpectedly as some of the risk factors associated with OH development, such as neuropathy, diabetes mellitus, heart disease, Parkinson’s disease, and need of polytherapy are more frequent in the elderly [16, 17]. POTS is also commonly diagnosed in adolescents, particularly within two years from the beginning of puberty, but often more benign in its course and often self-limiting [18]. POTS is two to three times more frequent in female adolescents compared to male and almost 90% of adults patients with POTS are female [18], with not showing a clear preference for the female sex in older adults. In our cohort we observed higher incidence of POTS and OH in males versus females. This was unexpected and not easily explained. Additional studies are needed to explore this apparent sex-based difference.

Treatments with antihypertensive agents is a well-recognized risk factor for OH and POTS [19]. In this retrospective analysis, we have not found statistically significant difference in age, sex, use of antihypertensive drugs, between patients with and without OH or POTS. We observed an association between radiotherapy and some classes of chemotherapeutic agents and the development of dysautonomia. However, none of these treatments maintained the statistical significance in the multivariate model, suggesting that other, yet unidentified, factors may contribute to OH and POTS incidence after transplant. Moreover, patients with hematologic malignancy are often treated with several chemotherapy drugs before the HCT, thus complicating the evaluation of the effect of the single agent. A previous study on breast cancer patients highlighted the association between cardiac autonomic dysfunction and anthracyclines [20], supporting a potential role of this class of drugs in OH and POTS development. Head and neck radiotherapy has been associated with a reduction of BP values after the treatment, but no OH has been recorded [21]. In another cohort of 282 cancer survivors referred to a cardio-oncology program, 22 subjects were diagnosed with autonomic dysfunction [22]. Of these, the majority had hematologic disorders and one third had undergone HCT [22]. The subjects showed impairment in multiple components of the autonomic function (sudomotor, adrenergic and cardiovagal) [22] [22]..

Given the timely association with HCT, it is possible that the HCT itself is the leading cause of OH and POTS in this group of patients. Indeed, the transplant lead to an important burden of inflammation, which may be the trigger for OH and POTS development [23]. The median time from HCT to diagnosis of OH or POTS was 82 (24–248) days. Further studies are required to better investigate the causes of OH and POTS after HCT.

OH is associated with diurnal BP variability and, often, with supine hypertension, which both contribute to intermittent increase in afterload, favoring the development of end-organ damage [5]. This observation was confirmed also in our cohort, in which 1/3 of patients who developed OH after the HCT presented also supine hypertension.

The management of OH and POTS is mainly driven by symptoms and non-pharmacological treatments are usually considered as first line therapy [5, 24]. The non-pharmacological treatments alone can be, however, insufficient in severe cases. One of the limitations of this analysis is the inability to capture the use of non-pharmacological treatments in terms of prescriptions and compliance. The majority (91%) of patients in our cohort required drug prescriptions for OH and POTS. Midodrine, a selective and direct a1-adrenoreceptor agonist, has shown to be effective in reduce symptoms and improve quality of life [25]. Fludrocortisone is an effective volume expander, but its use is contraindicated in patients with heart failure, kidney failure or supine hypertension [26]. In POTS, β-adrenergic blockers have been reported to improve symptoms [27, 28]. None of these treatments have however been proven to be superior compared to others in reducing outcomes and none of these treatments have been specifically studied in patients after HCT. In our experience midodrine was the most commonly used pharmacologic agent for OH, with fludrocortisone being added in non-responders, and β-adrenergic blockers being used in both patients with OH and POTS. The median duration of treatment was median duration of 120, although a third of patients remained on treatment long-term.

HCT survivors are at risk for several acute and chronic CV complications [29, 30]. OH and POTS, however, are rarely considered to be part of these possible adverse consequences, and patients may be not actively screened for this complication after transplant, therefore the exact incidence of these complications in unselected HCT recipients is unknown. OH and POTS can be debilitating, worsen the quality of life of the patients, and may delay the recovery following transplantation [31]. Several observations have linked OH to negative CV outcomes [32] and a recent meta-analysis confirmed the association between OH and the risk of all cause-death, coronary heart disease, heart failure and stroke [33].

Our results are particularly interesting because we describe for the first time the prevalence of OH and POTS in patients referred to cardio-oncology after HCT. The diagnosis of OH and POTS have been made by a cardiologist expert in the cardio-oncology field and confirmed with a careful chart review. Some limitations should be acknowledged. First, this is a retrospective study with obvious potential selection bias toward inclusion of patients with symptoms and/or at perceived cardiovascular risk, therefore limiting our ability to apply these findings to unselected HCT recipients. Second, the lack of a systematic assessment of vital signs may have led to inaccuracies in the detection of OH/POTS and calls for future prospective studies. Third, it is a single-center study, although conducted in a large hospital serving a diverse groups of patients. Finally, the limited number of cases did not allow us to evaluate the possible greater benefit of a treatment compared to the others.

## Conclusion

Orthostatic intolerance syndromes are commonly diagnosed in patients referred to the cardiologist after HCT, involving approximately 1/3 of patients and requiring pharmacological therapy to cope with symptoms in the majority of cases. Risk factors specific to this population have been identified but cannot fully explain the incidence of POTS and OH after HCT. Additional research is needed to define the actual incidence of OH and POTS after HCT, identify risk factors, and determine the most effective therapeutic strategy.

## Supplementary Information

**Additional file 1: Supplemental Figure 1**. Antineoplastic treatment before transplant. **Supplementary Table 1**. Chemotherapy/immunotherapy agents used in the overall cohort.

## Data Availability

The datasets analyzed during the current study are available from the corresponding author on reasonable request.

## Abbreviations

BP: Blood pressure
CI: Confidence interval
CV: Cardiovascular
CVD: Cardiovascular diseases
HCT: Hematopoietic cell transplantation
HR: Heart rate
OH: Orthostatic hypotension
OR: Odds ratio
POTS: Postural tachycardia syndrome
