# Supplementary material for: Orthostatic intolerance syndromes after hematopoietic cell transplantation: clinical characteristics and therapeutic interventions in a single-center experience
Source: Cardiooncology. 2021 Nov 30;7:40. doi: 10.1186/s40959-021-00126-7 (PMC8630909; doi:10.1186/s40959-021-00126-7)
Supplement: Supplementary file 1 — Additional file 1: Supplemental Figure 1. Antineoplastic treatment before transplant. Supplementary Table 1. Chemotherapy/immunotherapy agents used in the overall cohort. [file 40959_2021_126_MOESM1_ESM.docx]

**Supplemental Figure 1. Antineoplastic treatment before transplant.**

The column graphic represents the percentage of patients treated with radiotherapy or with chemotherapy/immunotherapy agents before the transplant.

Alkylating agents: Cyclophosphamide, Melphalan, Dacarbazine, Ifosfamide, Carboplatin, Oxaliplatin, Busulfan, Bendamustine, Carmustine, Cisplatin, Thiotepa.

Antimetabolites drugs: Gemcitabine, Cladribine, Cytosine, Fluorouracil, Clofarabine, Decitabine, Mercaptopurine, Hydroxyurea, Methotrexate, Azacytidine, Cytarabine, Fludarabine.

Anthracyclines: Doxorubicin, Idarubicin, Daunorubicin.

Epipodophyllotoxin: Etoposide

Anti-microtubules: Vinblastine, Vincristine, Vinorelbine, Paclitaxel.

Antitumor monoclonal antibodies: Rituximab, Blinatumomab, Daratumumab, Ibritumomab, Alemtuzumab, Obinutuzumab, Brentuximab.

Proteasome inhibitors: Bortezomib, Carfilzomib, Ixazomib.

Angiogenesis inhibitor: Lenalidomide.

Kinase inhibitors: Imatinib, Ruxolitinib, Ibrutinib, Nilotinib, Idelalisib, Ponatinib, Gilteritinib, Dasatinib.

Antitumor antibiotics: Bleomycin.

Histone-deacetylase inhibitors: Romidepsin, Vorinostat.

Anthracenedione: Mitoxantrone.

Retinoic acid derivative: Bexarotene.

**Supplementary table 1. Chemotherapy/immunotherapy agents used in the overall cohort.**

|  | **Patients (n=132)** |
| --- | --- |
| **Angiogenesis inhibitor** | 30 (22.7) |
| Lenalidomide | 30 (22.7) |
| **Retinoic acid derivative** | 1 (0.8) |
| Bexarotene | 1 (0.8) |
| **Histone-deacetylase inhibitors** | 6 (4.5) |
| Romidepsin | 1 (0.8) |
| Vorinostat | 5 (3.8) |
| **Corticosteroids** | 45 (34.1) |
| **Kinase inhibitors** | 21 (15.9) |
| Imatinib | 4 (3.0) |
| Ruxolitinib | 8 (6.1) |
| Ibrutinib | 5 (3.8) |
| Nilotinib | 1 (0.8) |
| Idelalisib | 1 (0.8) |
| Ponatinib | 1 (0.8) |
| Gilteritinib | 1 (0.8) |
| Dasatinib | 4 (3.0) |
| **Antimetabolites** | 89 (67.4) |
| Gemcitabine | 7 (5.3) |
| Cladribine | 1 (0.8) |
| Fluorouracil | 1 (0.8) |
| Clofarabine | 3 (2.3) |
| Decitabine | 3 (2.3) |
| Mercaptopurine | 1 (0.8) |
| Hydroxyurea | 11 (8.3) |
| Methotrexate | 10 (7.6) |
| Azacitidine | 21 (15.9) |
| Cytarabine | 51 (38.6) |
| Fludarabine | 50 (37.9) |
| **Monoclonal antibodies** | 7 (5.3) |
| Blinatumomab | 1 (0.8) |
| Daratumumab | 1 (0.8) |
| Ibritumomab | 1 (0.8) |
| Alemtuzumab | 1 (0.8) |
| Obinutuzumab | 1 (0.8) |
| Brentuximab | 6 (4.5) |
| **Anti-microtubules** | 39 (29.5) |
| Vinblastine | 6 (4.5) |
| Vincristine | 32 (24.2) |
| Vinorelbine | 1 (0.8) |
| Paclitaxel | 1 (0.8) |
| **Epipodophyllotoxin** | 39 (29.5) |
| Etoposide | 39 (29.5) |
| **Antitumor antibiotics** | 8 (6.1) |
| Bleomycin | 8 (6.1) |
| **Anthracenedione** | 3 (2.3) |
| Mitoxantrone | 3 (2.3) |
| **Anthracyclines** | 57 (43.2) |
| Doxorubicin | 37 (28.0) |
| Idarubicin | 15 (12.1) |
| Daunorubicin | 9 (6.8) |
| **Proteasome inhibitors** | 37 (28.0) |
| Bortezomib | 36 (27.3) |
| Carfilzomib | 6 (4.5) |
| Ixazomib | 1 (0.8) |
| **Anti-thymocyte globulin** | 57 (43.2) |
| **Alkylating agents** | 123 (93.2) |
| Cyclophosphamide | 64 (48.5) |
| Melphalan | 50 (37.9) |
| Dacarbazine | 6 (4.5) |
| Ifosfamide | 15 (12.1) |
| Carboplatin | 14 (10.6) |
| Oxaliplatin | 5 (3.8) |
| Busulfan | 51 (38.6) |
| Bendamustine | 12 (9.1) |
| Carmustine | 14 (10.6) |
| Cisplatin | 4 (3.0) |
| Thiotepa | 1 (0.8) |

The data are presented as a number and (%) of all cases.
